# Binasal Occlusion (BNO), Visual Motion Sensitivity (VMS), and the Visually-Evoked Potential (VEP) in mild Traumatic Brain Injury and Traumatic Brain Injury (mTBI/TBI)

**DOI:** 10.3390/brainsci7080098

**Published:** 2017-08-09

**Authors:** Kenneth J. Ciuffreda, Naveen K. Yadav, Diana P. Ludlam

**Affiliations:** 1 Department of Biological and Vision Sciences, State University of New York, College of Optometry, New York, NY 10016, USA; dianaeye@aol.com; 2 Chicago College of Optometry, Midwestern University, Downers Grove, IL 60515, USA; nyadav@midwestern.edu

**Keywords:** traumatic brain injury (TBI), visually-evoked potential (VEP), binasal occlusion (BNO), visual motion sensitivity (VMS), visuomotor, visual motion perception, magnocellular pathway, dorsal stream

## Abstract

The diagnosis and treatment of the possible visual sequelae in those with traumatic brain injury (TBI) represents an important area of health care in this special population. One of their most prevalent yet elusive visual symptoms is visual motion sensitivity (VMS). In this review, we present the basic VMS phenomenon and its related symptoms, clinical studies in the area, clinical research investigations using the visual-evoked potential (VEP) as a cortical probe, and possible mechanisms and related neurophysiology that may underlie VMS. Lastly, therapeutic interventions are briefly described, as well as future directions for clinical research and patient care in those with VMS and TBI.

## 1. Introduction

**Figure 1:**
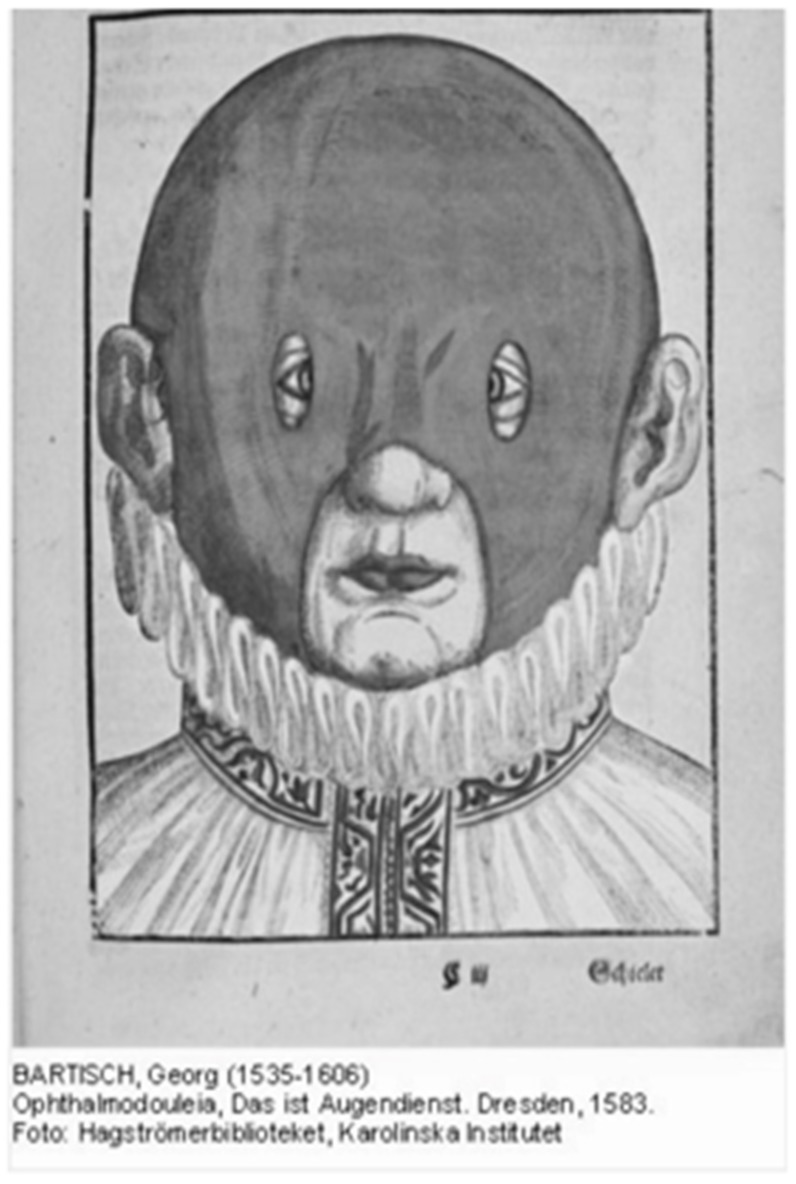
Strabismus mask for esotropia.

The use of binasal occluders (BNO) and bitemporal occluders (BTO) in medicine, in particular ophthalmology, has a long and interesting history for the treatment of strabismus (deviation of an eye from parallelism). Mention of such occlusion dates back to at least 600 AD or so ([1] for a review). Here, BTO was used as a treatment for exotropia (outward deviation on one eye). The concept was that, in those with exotropia, temporal occlusion would force them to converge their eyes to the median plane to attain binocular fixation and fusion. This is basically one of the first mentions of the clinical practice of “orthoptics”, the medically-based remediation of abnormal binocular vision, especially fusional problems related to strabismus [1]. Over the next millennium, it evolved further from small and simple occluders into a more encompassing “strabismus mask”, presumably to have better compliance despite its poor cosmesis, primarily for the treatment of esotropia (inward deviation of one eye) ([1] for a review) (Figure 1). Thus, strategic location of the occluders could be used for either esotropia or exotropia, and sometimes for hypertropia (vertical deviation of one eye) when placed in the vertical position. This basic concept continues today both in ophthalmology and optometry for strabismus, as well as other binocular vision disorders (e.g., spasm of the near reflex) [2], once again using simple occluders.

**Figure 2:**
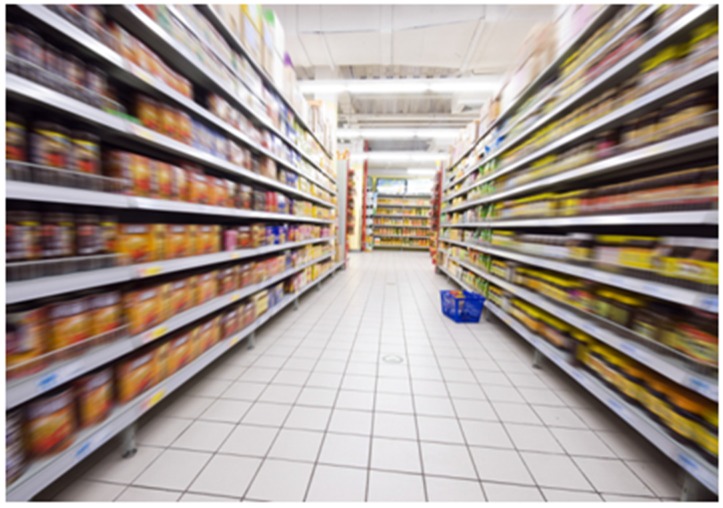
Gibsonian optic flow while walking down a supermarket aisle.

However, approximately 30 years ago, the employment of BNO in optometry took a new direction. It began to be used for the treatment of visual motion sensitivity (VMS) in brain-injured patients, typically those with mild traumatic brain injury (mTBI). VMS is a relatively common symptom in these patients, likely occurring in approximately 40% of the patients [3,4]. VMS refers to the occurrence of specific visually-related symptoms in response to a moving visual stimulus, especially encompassing the peripheral visual field which normally does not invoke these sensations in others. Such situations as the scrolling of a large computer screen, traversing a busy supermarket (“supermarket syndrome”) or a mall producing Gibsonian optic flow [5] (Figure 2), sitting in the back seat of a moving car, or riding an escalator, as well as other moving environments, may be provocative and therefore lead to the feeling of imbalance, dizziness, disorientation, poor spatial orientation, and even nausea (e.g., “car sickness”) [6,7,8,9].

In this review, we will cover both the clinical and laboratory-based studies involving BNO and VMS, with an emphasis on the objective, visually-evoked potential (VEP) findings. Both the clinical utility, as well as the possible underlying perceptual and neurological mechanisms, will be addressed. Future directions for research will also be considered.

### Methods

The following resources were used for the present review. They included computer searches using PubMed, Yahoo, Google, and Google Scholar. Search terms included “visual motion sensitivity”, “VMS”, “visual motion perception”, “binasal occlusion”, “BNO”, “vision in mTBI/TBI”, “brain injury”, “VEP”, and “magnocellular pathway”. In addition, relevant books, book chapters, and published papers in the area were used other than those found in our formal computer searches. 

## 2. Clinical Case Studies

Surprisingly, given the high prevalence and bothersome nature of VMS, there is a relative paucity of clinical studies and case reports in this area of mTBI/TBI, as well as in other forms of acquired brain injury (ABI) and neurological disorders (e.g., cerebral palsy, multiple sclerosis) [7,8]. Unfortunately, visually-evoked potential (VEP) recordings were not obtained, both with and without the BNO in place, in these clinical reports. However, the cases and their findings provide important insights into the VMS phenomenon and related symptomatology.

One of the earliest case reports in this area was that of Proctor (2009) in a 46-year-old male patient [9]. He had been involved in 13 motor vehicle accidents (MVA) over the prior 10 years. The patient later received a full general medical examination, as well as a neurological evaluation including the inner ear, with no clear cause found for his persistent visual symptoms. This examination was performed eighteen months prior to his optometric vision examination. His recent MRI was normal, as were the earlier ones that had been performed. However, his symptoms at the time of the initial, comprehensive optometric vision examination (including a visual field), and which had persisted over the prior ten year period, included VMS/”supermarket syndrome”, dizziness, balance and gait difficulty, especially in crowded environments, nausea when in a moving vehicle, and poor depth perception, all of which are common visually-based symptoms in mTBI [3,4,6]. He was prescribed a course of vision therapy (i.e., a sequence of specific remedial procedures and protocols involving the oculomotor system and other vision-related aspects incorporating the principles of perceptual and motor learning) [10], with an oculomotor emphasis, due to the poor fixation, pursuit, and saccadic abilities uncovered. At the sixth therapy session, BNO was attempted. The improvements were immediate as he walked down a long hallway and performed concurrent visuomotor tasks. The BNO was added to his home therapy; for example, he was instructed to use them when riding as a passenger in a car. The patient reported reduced symptoms in a motor vehicle, as well as further improved mobility with less symptoms, with the BNO. However, he did not use the BNO for driving, as he felt that the occluders restricted his peripheral visual field. 

More recently, Gallop (2014) presented two cases of mTBI in which BNO was found to be helpful [7]. The *first* patient was a 67-year-old male who was involved in an MVA. His initial visual symptoms included diplopia, pain in his right eye with ocular movement, and balance problems. An ophthalmological vision examination shortly after the MVA revealed diplopia for which base-out prisms were prescribed, but without any relief. Months later, he was examined by his optometrist of many years, and thus all of the MVA-related visual symptoms could be compared against those pre-injury. The patient reported the same visual symptoms as he did earlier to the ophthalmologist, but he now exhibited several new visual signs: reduced distance visual acuity, reduced stereoacuity at near, vertical phoria at near, and large exophoria at near. None of these symptoms and signs were present prior to the MVA. Due to his visual symptoms, especially related to balance, it was decided to try BNO on the patient. Immediately, his symptoms were markedly reduced, especially during ambulation. The BNO, along with the oculomotor-based vision therapy, were prescribed with continued success. The *second* patient was a young, female administrator who had sustained an mTBI one year earlier. She had hit the top of her head and was temporarily “stunned”. Her symptoms included reading difficulties, light sensitivity, discomfort on the computer, variable ocular focusing, spatial disorientation, and balance problems. All busy environments, including her office, triggered her balance and disorientation problems. The patient’s comprehensive optometric vision examination was unremarkable, and visual acuity was 20/20. However, she was nauseous for several hours after each section of the basic vision test battery, which is not uncommon in these patients. Thus, she elected not to perform any further vision testing and to forgo the tentatively suggested vision therapy. About two years later, the patient returned to the same optometric clinic with the same persistent visual problems. In the interim, she was being treated for anxiety, with some degree of intermittent relief of her visual symptoms. There were now fewer symptoms during the subsequent vision examination, and all testing could be completed. She was recommended a course of vision therapy. In addition, BNO was attempted. The effect of the added BNO was immediate, with about 80% reduction in her symptoms as she ambulated about the doctor’s office. The markedly reduced symptoms of VMS in busy environments, such as the street and her workplace, continued. The combination of the BNO and the vision therapy has allowed her to remain much less symptomatic under all naturalistic conditions over the several months of follow-up.

**Figure 3:**
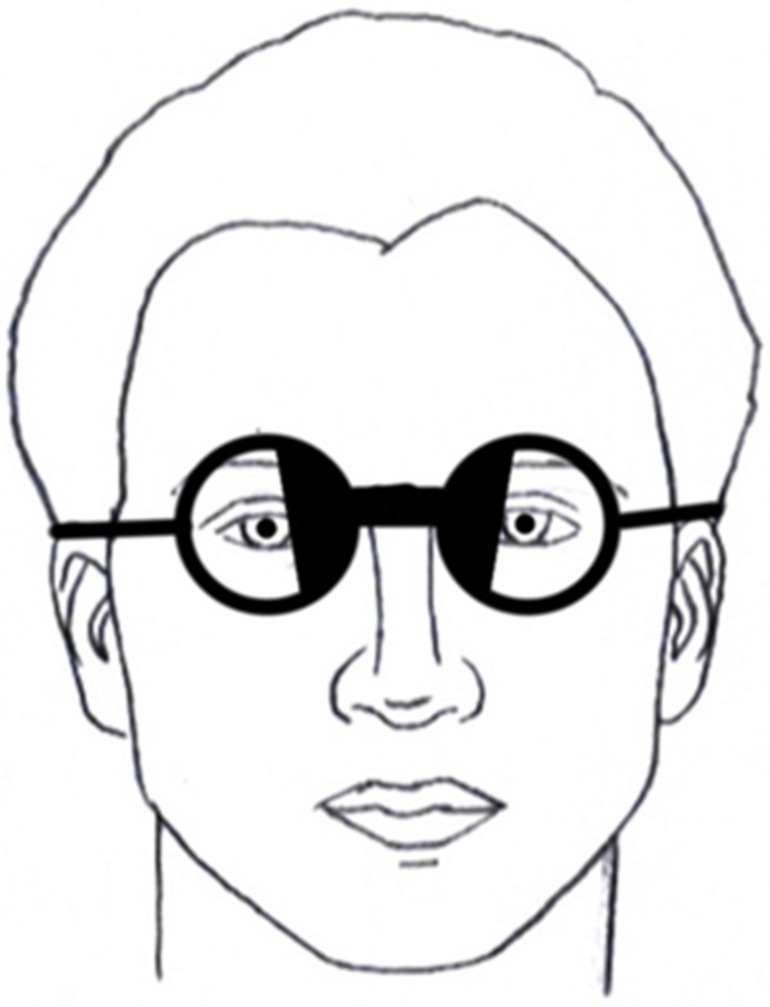
Schematic representation of binasal occluders on a subject [11,12].

Based on the above clinical studies, what might be the prevailing mechanism? A most intriguing suggestion was proposed by Gallop (1998) [8]. He speculated that the BNO, essentially functioning as an “object” in the patient’s environment, provided a stable “visual frame of reference” for these patients with symptoms such as dizziness and disorientation, as the BNO moved with head movement. That is, the patient now had a stable object in the visual field during all tasks which acted as a spatial-perceptual visual ‘anchor’. This may be even more true when the BNOs are oriented vertically, rather than angled as frequently done (Figure 3), to provide a more direct gravity-based reference. 

## 3. Laboratory Studies

Once again, and surprisingly, there have been a paucity of studies in clinical laboratories assessing the efficacy of BNO in mTBI/TBI. The first investigation was published nearly 20 years prior to the more recent studies. All three investigations included the VEP as the primary outcome measure.

### 3.1. Padula Group

The first investigation of BNO in mTBI/TBI was by Padula et al. (1994) [13]. This hospital-based study was conducted in ten adult (ages 22–46 years) patients with medically-documented TBI. The presence of VMS was not explicitly indicated, and thus not a prerequisite. All were ambulatory. They were compared with 10 normal adults (ages 23–46 years) from the hospital staff without any history of either TBI or VMS. None of the tested individuals had strabismus, as the VEP testing was always conducted under binocular-viewing conditions, and its presence would have contaminated the findings and their interpretation.

Following a comprehensive optometric vision examination, the VEP (Nicolet system, San Carlos, CA) testing commenced. Important details of the methodology are as follows. A central/near retinal periphery field, checkerboard pattern was used as the test stimulus. It had high-contrast with a check size of 30 minutes of arc and a stimulus pattern reversal rate of 1.9 reversals/s. An ABA experimental design was used: A = habitual distance refraction, B = habitual distance refraction plus the vertically-oriented, translucent tape BNO and 2 BI prisms before each eye, and then condition A again to assess for and assure a stable baseline. The primary VEP metric was its peak-to-peak amplitude (N1-P1), a standard VEP parameter. The VEP was completed in all ten normal controls and in nine of the ten individuals with TBI.

The VEP findings were as follows. In the normals, the VEP amplitude with the combined BNO/prisms increased in 6/10, decreased in 2/10, and remained the same in 2/10, with a range of −14% to +11%, and a mean decrease in amplitude of −0.41 microvolts, as compared to the baseline values. These small, mixed findings suggested a ‘noise’ phenomenon; that is, a lack of any consistent cortically-based physiological effect, as one might predict in this control population. In contrast, in the TBI group, the amplitude increased in 8/9 and decreased in 1/9, with a range of −5% to +54% (mean = +20%, +1.38 microvolts). There was a statistically significant effect of the BNO/prism combination in the TBI group versus the control group (*t*-test, *p* < 0.01). Some patients reported a reduction of symptoms (e.g., reduced visual instability) with the BNO/prism combination incorporated into their distance spectacle prescription. No other related testing was performed, such as formally assessing visuomotor ability or general ambulation with and without the BNO/prism combination. 

Two mechanisms were proposed, which were hypothesized to increase the effectiveness of the patient’s “binocular cortical cells”, with the BNO/BI combination. As described above in the ‘clinical studies’ section by Gallop (1998) [8], Padula et al. (1994) [13] also proposed that the BNO provided ‘structure’ to the patient’s overall visual field. They also proposed that the base-in prisms provided a degree of ‘field expansion’. Prisms do displace and deviate incident light per Snell’s Law [14], and they do so differentially as a function of its angle of incidence. This results in magnification of the visual field in a non-uniform manner, and thus some degree of ‘field expansion’ does take place. These proposed mechanisms remain speculative.

### 3.2. Ciuffreda Group

The next and most recent experiments in this area were performed in the Ciuffreda laboratory about 20 years later [11,12]. These two studies extended the earlier one by Padula et al. (1994) [13], with their primary goal being to disambiguate the effects of the BNO/BI prism combination, as was used in the Padula et al. (1994) study [13].

In the *first investigation* by Ciuffreda et al. (2013) [11], the question was simple: “What was the effect of BNO alone on adults with chronic mTBI and symptomatic VMS?” There were 10 patients with medically-diagnosed mTBI and VMS (mean age 28.9 years), and there were 10 matched, visually-normal (VN) controls (mean age 26.7 years) without VMS, with both groups having 20/20 or better corrected visual acuity in each eye. None had a history of seizures, strabismus, amblyopia, or any ocular, systemic, or degenerative neurological disease. All underwent a comprehensive optometric vision examination prior to laboratory testing.

**Figure 4:**
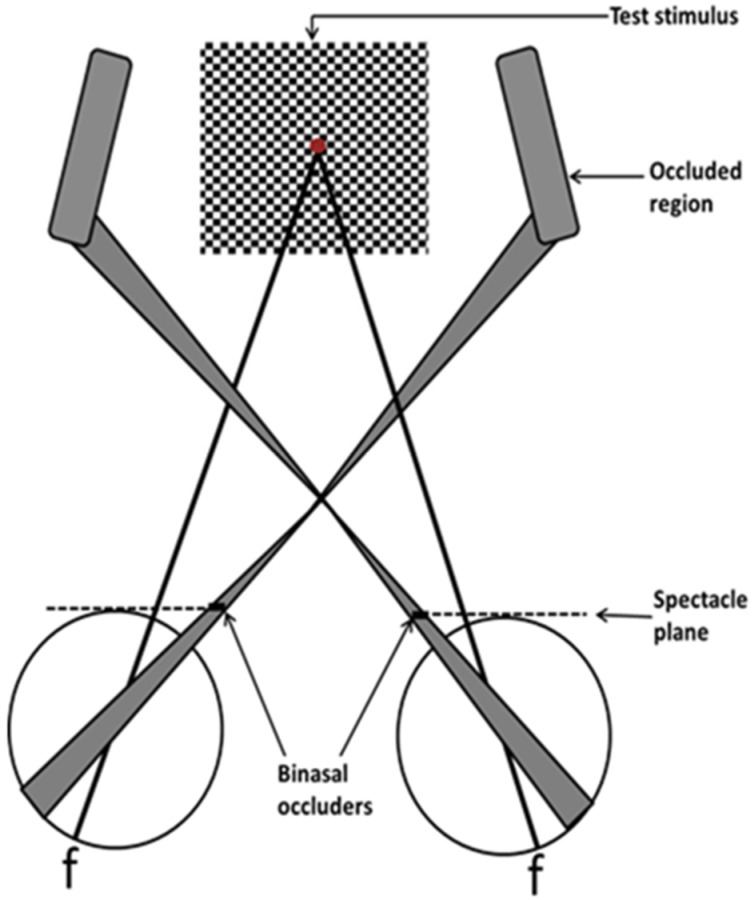
Representation of the binocular visual-field with binasal occluders in place, including the checkerboard visual stimulus. Not drawn to scale. f = fovea [12].

The VEP (DIOPSYS system, Pine Brook, NJ, USA) stimulus and test conditions were as follows. The test target consisted of a 17 H by 15 V degree, alternating (four reversals per second), black-and-white, checkerboard pattern (64 × 64, 20.6 min arc) placed one meter away along the subject’s midline. Luminance was 64 cd/m-squared with a contrast of 85%. The test duration was 20 s. Two trials per test condition (spectacles only or spectacles with obliquely-oriented, opaque black BNO, with binocular viewing) were averaged in each subject, and then averaged across each group, for the parameters of amplitude and latency. A schematic representation of a subject with the BNOs in place is presented in Figure 3. A schematic representation of the stimulus array with the subject and the BNOs in place is presented in Figure 4. The BNOs (5.7 H × 15 V degs) were cross-projected 5.5 degrees to either side of the stimulus array and occluded portions of the bitemporal retinas. 

**Figure 5:**
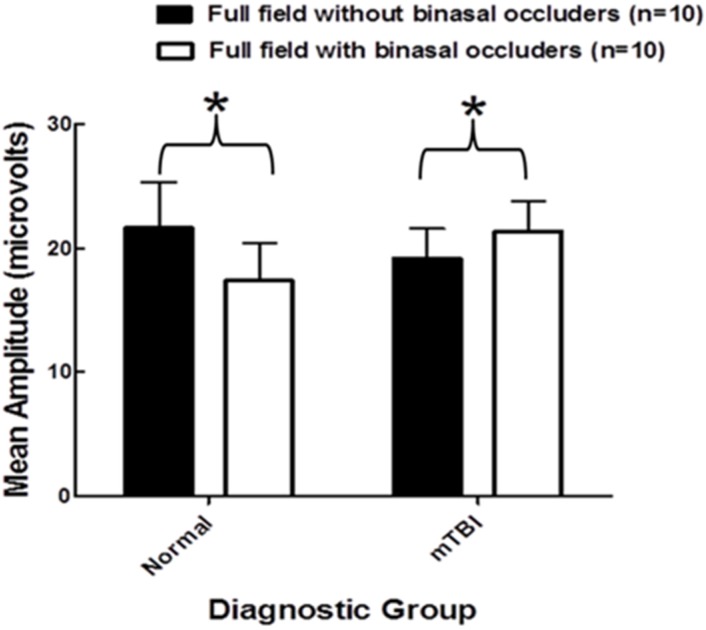
Group mean amplitude for the full field without and with binasal occluders in normals and in mTBI [12]. * means statically significant.

**Figure 6:**
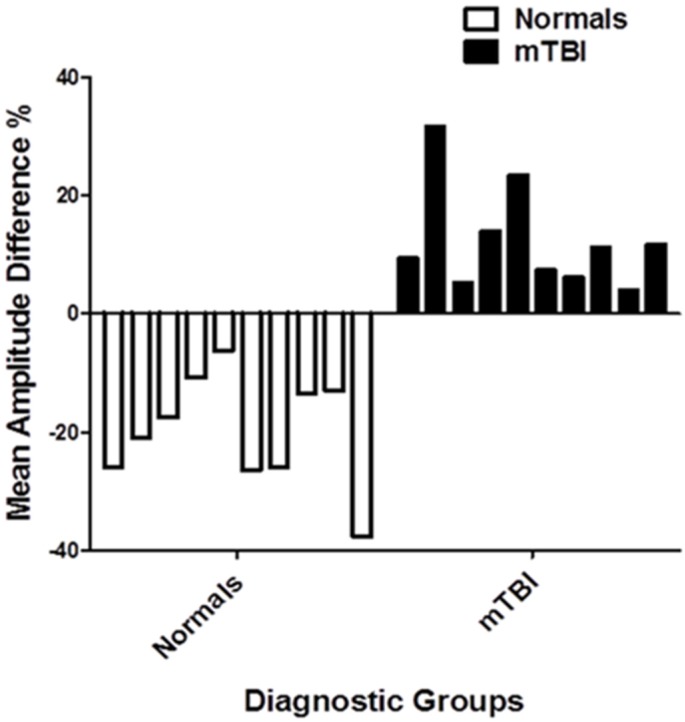
Diagnostic group mean amplitude difference for individual subjects (%). Positive and negative percentage values represent an increase or decrease in mean amplitude difference values, respectively [11].

The mean amplitude (microvolts) findings are presented in Figure 5. In the VN group without BNO, it was 21.6 with a range from 9.1–45.4; with the BNO, it was 17.4 with a range from 7.4–35.9. This *decrease* with BNO was found in all 10 subjects, which was statistically significantly (binomial test, *p* < 0.01) (Figure 6). In the mTBI group without BNO, it was 19.2 with a range from 12.2–35.5; with BNO, it was 21.3, with a range from 13.6–36.9. This *increase* with BNO was found in all 10 subjects (Figure 6), which was statistically significant (*p* < 0.01). These findings were repeatable 2–7 days later in a subset of subjects in each diagnostic group.

Latency was also assessed. It was within normal limits, and was not statistically different under any of the test conditions and diagnostic groups.

Lastly, the subjective impressions of both groups were queried. All VN subjects disliked the BNO. The blocked field was annoying and produced a sense of visual discomfort. In contrast, eight of the ten mTBI subjects with VMS liked the BNO (*p* < 0.01). They reported reduced symptoms (e.g., reduced nausea, reduced disorientation). Furthermore, they reported more accurate and comfortable ambulation, fixating of objects in the field, and grasping of objects. They also indicated that the VEP stimulus appeared clearer, brighter, and flickered less. In the other two mTBI/VMS subjects, they disliked the blocked field of the BNO, despite their objective cortical and subjective improvements with the BNO.

In the *second investigation* by Yadav and Ciuffreda (2014) [12], the question was a little more complex: “What were the independent and combined effects of the BNO *and/or* base-in prisms on adults with chronic mTBI and symptomatic VMS?” There were 15 patients with medically-diagnosed mTBI (mean age 35.2 years) with the symptom of VMS, and there were 20 matched, visually-normal (VN) controls (mean age 25.5 years) without the symptom of VMS, with both groups having 20/20 or better visual acuity in each eye. None had a history of seizures, strabismus, amblyopia, or any ocular, systemic, or degenerative neurological disease. All underwent a comprehensive optometric vision examination prior to laboratory testing.

The same VEP apparatus as described in detail in their earlier experiment above was used [11]. However, for each test condition in each subject, four trials were now averaged in each subject in each diagnostic group and then averaged across each diagnostic group. There were four test conditions, all with distance spectacle correction in place, performed in a counter-balanced manner to minimize order effects: (1) baseline, (2) BNO only before each eye, (3) two base-in prisms only before each eye, and (4) BNO plus two base-in prisms before each eye. The statistical analyses (ANOVA) revealed several important, significant findings (all *p* < 0.05).

**Figure 7:**
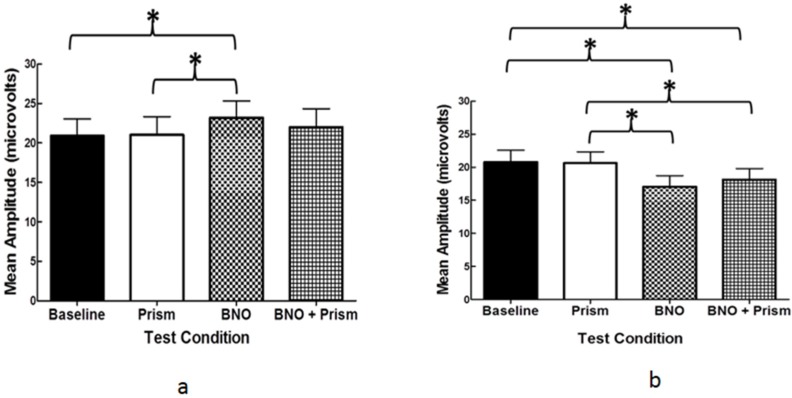
Group mean VEP amplitude for the four test conditions (baseline, prism, BNO, and BNO plus prism). Plotted is the mean +1 SEM. (**a**) visually-normal; (**b**) mTBI. Brackets with an asterisk (*) represent significant differences (*p* < 0.05) [12].

*VN* (*mean amplitude, microvolts*) (*Figure 7a*): Firstly, the baseline (20.8) and the prism only (20.6) conditions were not significantly different, which suggested that the prisms did not affect the VEP amplitude. Secondly, the BNO only condition (17.1) was significantly less than found in either the baseline (20.8) or the prism only (20.6) conditions. Thirdly, the BNO (17.1) and the BNO plus prism (18.1) conditions were not significantly different. Together, these findings suggested that the BNO condition alone was sufficient to have a significant effect on the VEP amplitude; the incorporation of the base-in prisms had no cortical advantage.

*mTBI* (*mean amplitude, microvolts*) (*Figure 7b*): Firstly, the baseline (20.9) and the prism only (21.0) conditions were not significantly different. Secondly, the BNO only (23.2) condition was significantly greater than either the baseline (20.9) or prism only (21.0) conditions. Thirdly, the BNO only (23.2) and the BNO plus prism (22.0) conditions were not significantly different. These findings suggested that the BNO condition alone was sufficient to have a significant effect on the VEP amplitude; the incorporation of the base-in prisms had no cortical advantage. 

**Figure 8:**
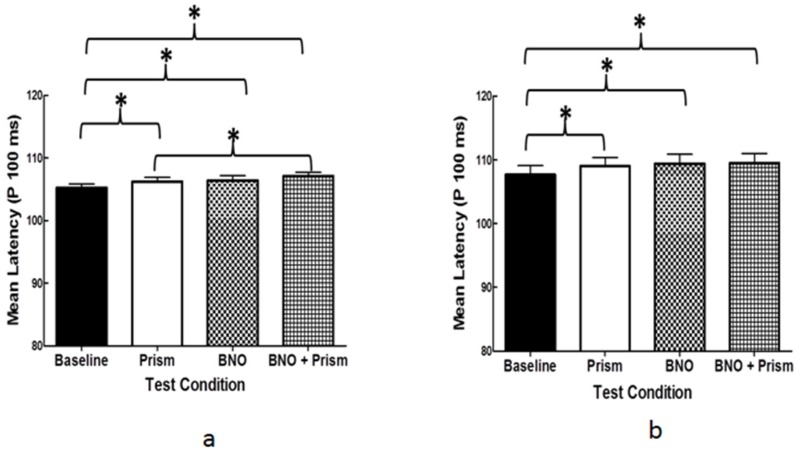
Group mean VEP latency (P100) for the four test conditions (baseline, prism, BNO, and BNO plus prism). Plotted is the mean +1 SEM. (**a**) visually-normal; (**b**) mTBI. Brackets with an asterisk (*) represent significant differences (*p* < 0.05) [12].

*mTBI* (*mean latency, milleseconds*) (*Figure 8a,b*): For both diagnostic groups, the mean latency was significantly increased for the BNO and/or prism conditions as compared to the baseline by a few milliseconds. This suggests small but significant increases in visual processing time for these optical conditions. However, all were within their laboratory and literature normative values.

**Figure 9:**
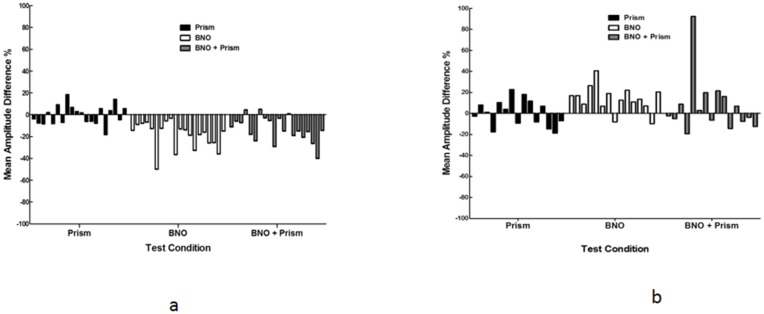
Percentage amplitude differences for three test conditions relative to baseline values for each subject. Negative values indicate a decrease in amplitude and positive values indicate an increase in amplitude. (**a**) Visually-normal; (**b**) mTBI [12].

The individual amplitude findings (percentage, %) are presented in Figure 9a,b. For the VN group, all showed a relative decrease in amplitude with the BNO only (range −49.0 to −3.2), nearly all decreased (17/20) with the BNO plus prism (range −39.5 to −4.9), and there was a mixed response with the prism only (10/20 decreased, range −18.2 to +18.4). The last result suggested a chance occurrence or ‘noise’ phenomenon, whereas the first two findings show the key BNO effect. For the mTBI group, all but two (13/15; *p* < 0.05) showed an increase in amplitude with the BNO only (range −9.7 to +40.6), there was a mixed result (7/15 increased) with the combined BNO plus prism (range −19.4 to +92.3), and again a mixed result (8/15 increased) with the prism only (range −18.8 to +22.7). Again, the mTBI findings revealed the uniqueness and consistent advantage of the BNO alone on cortical activity.

The VEP findings were repeated three weeks later in two subjects from each diagnostic group. The coefficient of variation (COV) statistic was used to assess this aspect. Values can range from 0.00 to 1.00, with smaller values indicating less variability, and hence better repeatability. Values ranged from 0.00 to 0.07, thus indicating an extremely good repeatability in these subjects.

Lastly, subjective testing was performed and compared under three visual and/or visuomotor conditions in those with mTBI: (1) viewing of either a blank wall, a stationary checkerboard pattern, or a flickering checkerboard pattern; (2) near grasping of objects; or (3) walking down a long corridor. For all conditions, most subjects (13/15; *p* < 0.05) reported the tasks to be best with the BNO, thus consistent with and confirming the objective VEP findings. Some of their comments are worth noting: most felt more stable, comfortable, and confident ambulating with the BNO; some commented on perceiving less “visual noise” especially in the periphery; and others indicated a sense of reduced sensory overload. Thus, there was a manifold of subjectively-based changes and improvements with the BNO present. Further research in this important area is warranted.

## 4. Proposed Mechanisms of BNO

Three possible perceptually-based mechanisms have been advanced to explain the VEP findings [11,12]. They are of particular clinical research interest.

The first proposed the presence of a “faulty spatial-temporal filtering mechanism” with regard to visual motion in the visual field [15]; that is, normals have the ability to neurally disregard/filter out all or most of the irrelevant visual motion information, especially in the periphery, while those with mTBI and VMS cannot. The latter may have an inability to neurologically inhibit this information either fully, or as needed, from entering their abnormal motion-based, visual processing stream (i.e., magnocellular pathway; dorsal stream), thus resulting in symptomatic VMS. The addition of the BNO in these patients may act to reduce the amount of the abnormal, neurological inhibition required, as parts of the bitemporal retinas are occluded, thus resulting in a relative increase in the VEP amplitude via this “disinhibition” mechanism, as found in all three laboratory experiments [11,12,13].

The second involves the “spread of suppression”, as commonly found in both strabismics [16] and in visually-normal individuals [17]. That is, any cortically-based suppressive effect spreads beyond the regions of the initially-directed suppression, and thus extends to contiguous regions of the visual field and correlated retinal regions by up to several degrees. Individuals with mTBI and VMS chronically attempt to suppress/inhibit the peripheral visual motion to reduce the problematic visual motion stimuli. However, since the VMS still persists, this suppression appears to be deficient/”faulty”, leading to only a partial suppression of the offensive visual motion information. This abnormal peripheral inhibitory effect interacts with the central field cortical excitation via the spread of suppression, thereby resulting in a net reduction the VEP response amplitude. However, with BNO added, portions of the habitually-suppressed field and correlated retinal regions are now occluded, thus resulting in reduction in the amount of suppression needed, and hence a relative increase in the VEP amplitude occurs, again as found in all three experiments [11,12,13]. The opposite was proposed for the VN group.

The third mechanism may be related to visual attention. Assuming that the BNO reduces some of the irrelevant and distracting peripheral visual motion information from the occluded bitemporal retinal regions, then attentional weighting would be shifted back to the central visual field to some extent. Increased visual attention has been demonstrated to increase the VEP amplitude in persons with mTBI [18]. Therefore, with the introduction of the BNO in those with mTBI and VMS, an enhancement of the centrally-mediated visual attention is believed to occur, which in turn would increase the VEP amplitude.

## 5. Neurophysiology Underlying BNO

The neurophysiology related to visual motion sensitivity is continuing to emerge and is rapidly evolving [19,20]. The parvocellular pathway (P-cell) is believed to be involved in the processing of fine detail visual information, generally associated with the fovea; it can be disregarded for the present discussion. In contrast, the magnocellular pathway (M-cell) is believed to be involved in the processing of general visual motion information typically associated more with the retinal periphery. The M-cell pathway projects to the visual cortical area V5, the medial temporal area (MT), and the medial superior temporal (MST) cortex, where general aspects of visual motion occurring in the visual field are integrated. It has been suggested that an mTBI/TBI would cause damage to the M-cell pathway, therefore adversely affecting visual motion sensitivity and perception of visual motion in the environment, hence leading to VMS [19,20]. The addition of the BNO would effectively reduce some of the abnormal visual motion perceived by the patient with mTBI and VMS, thus reducing their symptoms.

However, over the past few years, new and exciting research has suggested a more complex scenario, which may better explain the phenomenon and symptoms of VMS in mTBI/TBI. It involves the newly-discovered V6/V6a cortical areas in both monkey and man [21,22,23]. Extensive neurophysiological and brain imaging studies have suggested that these two areas are involved in the perception and analysis of optic flow and “self-motion/egomotion” across the entire visual field, as well as object motion. Thus, combining the older and newer concepts, the following scenario has been proposed [21,22]. The V5/MT/MST areas are involved in the analysis and early processing of visual motion signals with regard to their direction and speed, especially in the central visual field. In contrast, cortical areas V6 and V6a are involved in the analysis and processing of both visual object motion and self-motion across the entire visual field. It is these latter neural areas that we believe are particularly relevant to VMS. The authors hypothesized that V6/V6a can subtract, or parse out, signals related to object motion in the visual field from signals related to self-motion in the visual field. If this difference signal is accurate, and there is a match, then visual stability ensues. However, if this difference signal is not accurate, and there is a mismatch, then visual instability may ensue. We speculate that this is what may happen in the patient with mTBI and VMS following damage to the V6/V6a and related cortical areas involved in motion perception. This idea awaits scientific confirmation, but until then, it may be a reasonable conceptualization of the problem in these patients. The addition of the BNO, which occludes a region of the retinal periphery in each eye, would in effect act to reduce this signal difference or mismatch by reducing the amount of visual field activated, thus resulting in reduced VMS as found both clinically and in the laboratory studies.

Clearly, this is fertile territory for future investigations. This might include clinical and basic experiments in visual perception, brain imaging, and visuomotor activities.

## 6. Treatment

**Figure 10:**
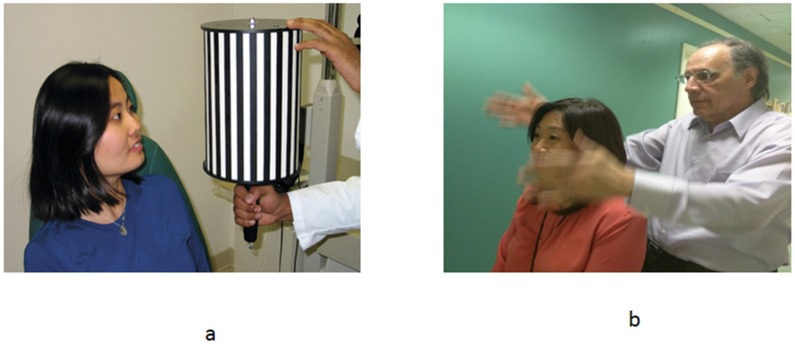
Visual motion habituation training: (**a**) optokinetic drum (OKN); (**b**) hand motion.

Three types of treatment have been proposed in the mTBI/TBI group with VMS [3,4]. The first is the application of BNO, as described earlier in the paper. The second is the prescription of tinted spectacle lenses. These can be either achromatic (i.e., neutral gray) or chromatic (e.g., reddish-blue). The idea here is to reduce the luminous intensity of the offensive stimulus, so that it becomes less effective and therefore less provocative to the patient’s abnormal visual motion processing system. Thirdly, there is visual motion habituation training (Figure 10a,b). This involves the use of either a slowly moving optokinetic (OKN) drum or the therapist’s moving hands, positioned to the side of the patient’s head, thus filling much of the peripheral visual field to simulate Gibsonian optic flow [5], with the therapist standing behind the patient. One would start by presenting the stimulus at a low velocity, and as the patient appears to be less symptomatic by its presence, the stimulus velocity/hand motion can be increased, et cetera [24]. This can also be performed with the OKN drum in the central field close to the patient’s face, so that much of the visual field in encompassed. The goal here is not to stimulate an OKN eye movement per se, which may occur at times, but rather to simulate visual motion (e.g., Gibsonian optic flow) in the patient’s visual field similar to what they may experience in real-life conditions (e.g., a long, crowded hallway). Lastly, Gibsonian optic flow can be presented in the clinic using computer-generated, wide-field, dynamic stimulation [25].

## 7. Study Limitations and Future Directions

There were three primary study limitations. First, sample sizes were relatively small. Second, the symptom of VMS was only ascertained from the standard clinical case history, and not by using a formal symptom rating-scale questionnaire for the perceptual problem of VMS and related aspects. Third, there was no long-term follow-up to assess the effect of the BNO treatment over time. 

There are several possible future directions for this important area of clinical and basic research. First, the application of BNO should be assessed in the clinic and laboratory environments in a larger sample of those with mTBI and VMS. Given the potential for helping this subset of patients, and the paucity of potential treatments, a clinical trial is warranted. It should include those with mTBI, both with and without the symptom of VMS, for comparative and control purposes, as well as for a better understanding of the possible mechanisms. Second, any new study should include the use of several visual and visuomotor tests/tasks to assess quantitatively the effects of BNO on visual performance. Third, since balance and spatial disorientation are common visually-based symptoms in these patients, the use of dynamic posturography would be critical to determine the effect of BNO on this complex process and the related quantitative metrics. This could be performed with a simple non-moving environment, as well as with more complex moving environments producing Gibsonian optic flow patterns per naturalistic situations (e.g., the supermarket). Fourth, testing should be extended to the moderate and severe TBI populations using the VEP and simpler vision and visuomotor testing/tasks. Fifth, in all types of TBI, the impact on quality of life (QOL) needs to be assessed to determine the positive effects on specific visual demands and tasks, both vocational and avocational. Sixth, and lastly, brain imaging should be performed to determine the site(s) of activity unique to the application of BNO in all categories of TBI.

## 8. Clinical Pearls

- BNO is a non-invasive method and is easy to apply in patients with mTBI and VMS.
- BNO seems to be an effective treatment method in patients with mTBI in reducing the symptoms caused by increased VMS.
- BNO provides immediate and sustainable effects in patients with mTBI and VMS.
- BNO seems to have a positive visuo-cortical effect in patients with mTBI and VMS.
- Using this simple clinical technique would be beneficial in treating these patients and helping them in performing their activities of daily living (ADL).

## 9. Conclusions

The use of BNO in patients with mTBI should be fruitful in future investigations. The clinical and laboratory data thus far show promise in the mTBI population with symptomatic VMS. While the possible mechanisms remain somewhat unclear and elusive, this should not prevent the clinician from attempting this approach, and related ones, in these patients. The clinician’s goal should be to provide symptom reduction and a better quality of life, which frequently will occur with the prescription of BNO.
